# Self-reported dyspnea and interest in a respiratory muscle training program among callers to the New York State Quitline

**DOI:** 10.18332/tid/196755

**Published:** 2025-01-29

**Authors:** Andrew D. Ray, Ellen M. Carl, Andrew J. Hyland, Mary E. Reid, Martin C. Mahoney, Christine E. Sheffer

**Affiliations:** 1 Department of Cancer Prevention and Control, Roswell Park Comprehensive Cancer Center, Buffalo, United States; 2 Department of Health Behavior, Roswell Park Comprehensive Cancer Center, Buffalo, United States; 3 Department of Cancer Screening, Survivorship and Mentorship, Roswell Park Comprehensive Cancer Center, Buffalo, United States; 4 Department of Internal Medicine, Roswell Park Comprehensive Cancer Center, Buffalo, United States

**Keywords:** dyspnea, smokers, respiratory muscle

## Abstract

**INTRODUCTION:**

Cigarette smoking is an important risk factor in the development of dyspnea. Programs designed to strengthen the respiratory muscles can improve dyspnea in people with or without lung disease. As a first step in understanding the feasibility of offering a respiratory muscle training (RMT) program to people who are seeking help to try to quit smoking, we asked callers who contacted the New York State Quitline about their dyspnea and potential interest in a home-based RMT program.

**METHODS:**

Consecutive callers who contacted the New York State Quitline (n=1019) between 19 May and 9 June 2023 completed the Modified Medical Research Council (mMRC) dyspnea scale and reported their level of interest in RMT. Participants were categorized as: high breathlessness (HB: 0–1), or low breathlessness (LB: 2–4). We examined characteristic differences between participants who reported HB versus LB and examined differences in level of interest in home-based RMT.

**RESULTS:**

Those with HB were older [mean (SD): 61.3 (12.5) vs 53.6 (15.0) years, p<0.001], had more cumulative years of smoking [38.8 (15.1) vs 28.8 (15.4) years, p<0.001], smoked more cigarettes per day [19.3 (10.5) vs 17.3 (8.8), p<0.01], reported more disability (p<0.001) and chronic health conditions (78.5% vs 53.9%, p<0.001). Those with HB also expressed greater interest in RMT [7.8 (3.3) vs 6.2 (4.1), p<0.001].

**CONCLUSIONS:**

These preliminary findings suggest that about 20% of quitline callers report clinically significant levels of breathlessness and most respondents, regardless of their level of breathlessness, report interest in a home-based RMT program, underscoring a potential opportunity to offer this program along with cessation support.

## INTRODUCTION

Cigarette smoking is an important risk factor in the development of respiratory symptoms including dyspnea^1^. According to the American Thoracic Society (ATS), dyspnea is defined as a subjective experience of breathing discomfort that consists of qualitatively distinct sensations that vary in intensity^2^. In current or former smokers, dyspnea is associated with poor health-related quality of life (QoL), cardiorespiratory fitness, physical inactivity, anxiety/stress, and increases in morbidity and mortality^3^. Recent reports also highlight that approximately 50% of people who smoke cigarettes (current and former) without clinically diagnosed lung disease have worse exertional dyspnea, as well as poorer QoL and exercise tolerance when compared to healthy non-smoking controls^4^. In fact, exertional breathlessness may be an early sign or symptom of chronic obstructive lung disease (COPD)^5^.

Traditional pathogenic mechanisms contributing to dyspnea in smokers with and without COPD include, amongst others, a reduction in lung elasticity, expiratory flow limitation, airway inflammation, infection, and genetic susceptibility^1^. Moreover, for a given ventilatory requirement, people who smoke cigarettes (current and former) require more inspiratory effort to overcome airflow limitation, thus increasing diaphragm activation and the use of accessory muscles to preserve the ventilatory response to exercise; ultimately this is perceived as greater dyspnea^6^. However, maintaining inspiratory pressure is difficult because cigarette smoking alters diaphragm structure and function, affecting its ability to generate force^7,8^. Because dyspnea represents an imbalance between the demand to breathe and the ability to breathe, the sensation can worsen with respiratory muscle weakness.

People who smoke cigarettes experience a ‘vicious dyspnea-inactivity cycle’ that limits inspiratory capacity, physical activity, and exercise performance, resulting in deconditioning and more dyspnea^9^. Programs designed to strengthen the respiratory muscles improve lung volumes, cardiorespiratory fitness, dyspnea, fatigue, anxiety/stress, and QoL in individuals with and without lung disease^10-12^. Inspiratory muscle training in patients with COPD improves respiratory muscle strength and endurance, dyspnea, and exercise stamina in conjunction with reduced activation of the diaphragm muscle^13^. In adults, a 4-week inspiratory muscle training study in people who currently (n=16), and never (n=16), smoked cigarettes, and a placebo control group (n=10) demonstrated significant improvements in expiratory muscle strength (21 ± 9 cmH_2_O vs 12 ± 5 cmH_2_O) and lung function [forced expiratory volume in 1 s (FEV1), slow vital capacity (SVC), and maximum voluntary ventilation in 12 s (MVV)]. The improvements following inspiratory muscle training were greater in those who smoked versus those who did not^14^. These findings suggest respiratory muscle training (RMT) may represent a non-pharmacological approach to reducing respiratory symptoms and increasing activity levels among current and former smokers.

Quitlines, available across the United States and in many other countries, offer free, accessible, evidence-based treatments for nicotine addiction via phone- and text-based platforms, making quitlines the largest existing tobacco treatment network^15-17^. Many people who contact quitlines are interested in seeking assistance with nicotine addiction and might also be interested in discussing methods to improve dyspnea symptoms via respiratory muscle training. As a first step in understanding the feasibility of offering an RMT program to people who are seeking help to try to quit smoking, we asked callers who contacted the New York State Quitline about their dyspnea and potential interest in a home-based RMT program.

## METHODS

### Design and procedure

This study employed a cross-sectional design. Consecutive callers to the New York State Quitline were assessed for dyspnea and interest in an RMT as part of the routine assessment conducted prior to callers being engaged in treatment for cigarette smoking.

### Participants

Those included in the study were English-speaking people seeking to quit smoking cigarettes who contacted the New York State Quitline between 19 May and 9 June 2023.

### Measures

The routine assessment administered to all callers included standard sociodemographic (sex, age, race/ethnicity, education level, disability status, and health insurance) and tobacco use items. Tobacco product use was examined by asking what types of tobacco products the participant used, number of years used, use of menthol products, and past quit attempts. Alcohol and cannabis use were assessed by asking how many days in the past 30 days alcohol or cannabis was used. Mental health was measured in two ways. One question asked whether a doctor had ever diagnosed the individual with a substance use disorder, anxiety disorder, bipolar disorder, or depression. The six-item Kessler psychological distress scale (K6) was also used to assess past 30-day psychological distress^18^. A K6 score ≥5 and <13 is indicative of moderate psychological distress^19-21^ and ≥13 is indicative of severe psychological distress^21^. Chronic health conditions were assessed by asking if a doctor had ever told them that they had asthma, cancer, diabetes, pre-diabetes, emphysema, heart disease, hypertension, kidney disease, or stroke.

This study added two additional questions to the pre-treatment intake assessment. The first question comprised the Modified Medical Research Council (mMRC) dyspnea scale^22^, a concise, valid method of assessing the degree of functional disability due to dyspnea or shortness of breath^23-26^. The mMRC asks respondents to choose one of five statements that best describes their shortness of breath; responses are scored on a scale from 0 to 4 (Table 1). According to the Global Initiative for Chronic Obstructive Lung Disease (GOLD) guidelines^27^, an mMRC score of ≥2 represents a threshold for separating LB from HB^28^, although patients with mMRC <2 may still have respiratory symptoms^28^. The second question assessed interest in a home-based RMT program by asking: ‘On a scale from 0–10, where 0=not at all and 10=the most ever, how interested would you be in a program to strengthen your respiratory muscles that you could do at home?’.

**Table 1:** Medical research council dyspnea scale grades

*Grade*	*Degree of breathlessness related to activities*
0	I only get breathless with strenuous exercise
1	I get short of breath when hurrying on a level ground or walking up a slight hill
2	I walk slower than other people of the same age on a level ground because of breathlessness or have to stop for breath when walking at my own pace on a level ground
3	I stop for breath after walking about 100 m or after a few minutes on a level ground
4	I am too breathless to leave the house or I am breathless when dressing

The table presents the mMRC dyspnea scale^28^. Patients were required to choose one answer that best describes their level of dyspnea. An mMRC score of ≥2 represents a threshold for separating low from high breathlessness^34^.

### Data analysis

Descriptive statistics were used to characterize participants. Responses to the mMRC dyspnea scale were categorized into two groups consistent with the GOLD guidelines^27^: Those who scored 0 or 1 were categorized as LB and those with scores of 2, 3, or 4 were categorized as having HB. Tests of significance, including chi-squared and one-way analysis of variance (ANOVA), were used to examine differences in participant characteristics between those categorized as LB and HB. The association between level of dyspnea (0–4) and level of interest in respiratory training (0–10) was examined using a Pearson correlation coefficient.

General linear main effects and exploratory full factorial models were used to examine differences in level of interest in RMT among participants with LB versus HB accounting for sex, age, race, education level, and Medicaid status. The significance level was set at alpha=0.05. All analyses were 2-tailed. IBM SPSS Statistics version 28.01 was used to analyze the data.

## RESULTS

### Respondent characteristics

During the 3-week data collection period, 1019 of 1044 (97%) consecutive callers to the New York State Quitline completed the routine pre-treatment assessment, the mMRC, and the interest in RMT. Approximately 20% of respondents reported an mMRC dyspnea score ≥2 and were categorized as HB. Participants were predominantly White (63.7%) and had smoked an average of 17.7 (SD=9.2) cigarettes per day for an average of 31 years (Table 2).

**Table 2:** Patient demographics grouped by level of dyspnea according to the mMRC (N=1019)

*Variables*	*All* *% (n)*	*Low breathlessness* *(0–1)* *% (n)*	*High breathlessness* *(2–4)* *% (n)*	*p*
**Total**	100 (1019)	79.9 (814)	20.1 (205)	
**Age** (years), mean (SD)	55.1 (14.8)	53.6 (15.0)	61.3 (12.5)	**<0.001**
**Sex**				
Male	45.0 (459)	44.1 (359)	48.8 (100)	0.239
Female	55.0 (560)	55.9 (455)	51.2 (105)	
**Race/ethnicity**				
White	63.7 (587)	63.7 (469)	63.4 (118)	0.808
Black	16.4 (151)	16.3 (120)	16.7 (31)	
Hispanic	13.8 (127)	13.5 (99)	15.1 (28)	
Other	6.1 (57)	6.5 (48)	4.8 (9)	
**Education level**				
≤ High school diploma	51.8 (454)	50.5 (353)	56.7 (101)	0.153
> High school	48.2 (423)	49.5 (346)	43.3 (77)	
**Medicaid**				
Yes	43.7 (445)	43.1 (351)	45.9 (94)	0.265
No	56.3 (574)	56.9 (463)	54.1 (111)	
**Smoking**				
Cigarettes/day	17.7 (9.2)	17.3 (8.8)	19.3 (10.5)	**0.006**
Years of smoking	30.9 (15.8)	28.8 (15.4)	38.8 (15.1)	**<0.001**
Pack-years	27.8 (21.0)	25.4 (19.4)	37.3 (24.3)	**<0.001**
Ever tried to quit before (yes)	84.8 (864)	84.2 (685)	87.3 (179)	0.154
Menthol use (yes)	47.2 (243)	47.3 (194)	46.7 (49)	0.497
**Chronic disease**				
Any	58.9 (600)	53.9 (439)	78.5 (161)	**<0.001**
Asthma	13.9 (142)	11.5 (94)	23.4 (48)	**<0.001**
Cancer	6.8 (69)	5.8 (47)	10.7 (22)	**0.011**
Diabetes	13.9 (142)	11.2 (91)	24.9 (51)	**<0.001**
Pre-diabetes	5.3 (54)	4.8 (39)	7.3 (54)	0.105
Emphysema	21.9 (223)	17.0 (138)	41.5 (85)	**<0.001**
Heart disease	7.6 (77)	5.9 (48)	14.1 (29)	**<0.001**
Hypertension	31.4 (320)	28.9 (235)	41.5 (85)	**<0.001**
Kidney disease	2.0 (20)	1.4 (1.1)	4.4 (9)	**0.010**
Stroke	2.6 (26)	2.0 (16)	4.9 (10)	**0.023**
**Mental health**				
Any	45.6 (464)	43.9 (357)	52.2 (107)	**0.020**
Alcohol or drug abuse	8.6 (88)	8.1 (66)	10.7 (22)	0.147
Anxiety	31.3 (319)	31.4 (255)	31.2 (64)	0.520
Bipolar	10.0 (102)	9.2 (75)	13.2 (27)	0.063
Depression	28.1 (286)	26.9 (219)	32.7 (67)	0.062
Schizophrenia	4.1 (42)	4.1 (33)	4.4 (9)	0.479
**Disability category**				
Any	41.5 (410)	35.5 (279)	65.2 (131)	**<0.001**
Blind	7.6 (75)	6.2 (49)	13.1 (26)	**0.002**
Deaf	7.7 (76)	6.5 (51)	12.5 (25)	**0.005**
Forgetfulness	20.5 (201)	18.0 (141)	30.3 (60)	**<0.001**
Difficulty walking	27.3 (269)	20.2 (159)	55.0 (110)	**<0.001**
Difficulty bathing	7.2 (71)	4.5 (35)	18.0 (36)	**<0.001**
Difficulty doing errands	9.6 (95)	5.9 (46)	24.6 (49)	**<0.001**
**K6 psychological distress**				
Total score, mean (SD)	5.3 (5.2)	5.0 (4.9)	6.6 (6.0)	**<0.001**
Mild (0–4)	52.7 (461)	54.9 (383)	44.3 (78)	**0.002**
Moderate (5–12)	36.8 (322)	36.4 (254)	38.6 (68)	
Severe (≥13)	10.4 (91)	8.7 (61)	17.0 (30)	
**Days of use in past month**				
Alcohol	2.8 (6.9)	3.0 (7.2)	2.0 (6.6)	0.081
Cannabis	3.2 (8.6)	3.5 (9.0)	1.9 (7.0)	**0.006**
**Interest in RMT** (0–10)	6.5 (4.0)	6.2 (4.1)	7.8 (3.3)	0.085

Low breathlessness (LB): mMRC score <2/4. High breathlessness (HB): mMRC score ≥2/4. K6: Kessler psychological distress scale; higher score equals worse psychological distress. RMT: respiratory muscle training. Tests of significance, including chi-squared and one-way analysis of variance (ANOVA), were used to examine differences in participant characteristics between those categorized as LB and HB.

### Differences between participants with HB (≥2 mMRC) and LB (<2 mMRC)

Participants with HB were significantly older (61.3 ± 12.5 vs 53.6 ± 15.0 years, p<0.001), smoked more cigarettes per day (19.3 ± 10.5 vs 17.3 ± 8.8, p<0.01), and smoked cigarettes for more cumulative years (38.8 ± 15.1 vs 28.8 ± 15.4, p<0.001). Participants with HB reported more disability (65.2% vs 35.5%, p<0.001), more chronic health conditions (78.5% vs 53.9%, p<0.001), and more mental health concerns (52.2% vs 43.9%, p<0.05). Participants with HB used cannabis for significantly fewer days in the past month than those with LB (1.9 ± 7.0 vs 3.5 ± 9.0 days, p<0.01). There were no between-group differences in sex, race, education level, Medicaid recipient status, past month alcohol use, or interest in RMT (Table 2).

### Interest in home-based training to improve lung health

**Figure 1:**
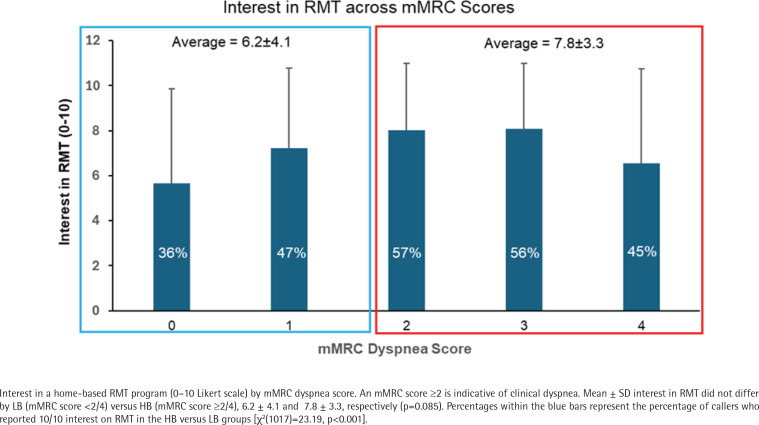
RMT interest levels by dyspnea category

Most participants reported moderate to strong levels of interest in a home-based RMT program (Figure 1). On a scale from 0 to10, the mean interest level in all participants was 6.5 (SD=3.9) and the median interest level was 8; 43% of all participants (n=437) reported an interest level of 10/10 and 30.1% (n=745) reported levels of interest between 4 and 9. The percentage of callers reporting a 10/10 interest in a home-based RMT program in the LB versus HB group was 40.0% and 54.1%, respectively [χ^2^(1017)=13.28, p<0.001]. Level of interest was also positively correlated to breathlessness severity [r(1017)=0.19, p<0.001]. The main effects GLM revealed that participants with HB reported greater interest in RMT than participants with LB [7.8 (SD=3.3) vs 6.2 (SD=4.1), F(1849)=18.61, p<0.001]. This significant difference was not found in the full factorial GLM where all potential interactions were included in the model [F(1849)=1.77, p=0.184]. Two interactions found to be significant in this model included an interaction between LB vs HB, sex, and education level, and an interaction between race, education level, and Medicaid status.

## DISCUSSION

A large proportion of people who are seeking treatment for smoking cessation with and without clinically significant dyspnea are interested in a home-based RMT program. The level of interest did not vary among those with LB and HB.

Individuals experiencing HB were characterized by older age, a higher prevalence of disability, chronic illness, mental health concerns as well as increased exposures based on years smoked and pack-years of use. Home-based RMT programs represent an easy-to-implement, non-pharmacological approach to improve smoking-related symptoms, performance, and QOL. While a limited number of RMT trials have enrolled people who smoke cigarettes, one study has reported that respiratory muscle strength improved more in people who smoked cigarettes compared to those who did not^14^. Although not resistance training, breathing exercises (blowing up a balloon and using breathing feedback) in elderly individuals who smoke cigarettes also significantly improved slow and fast vital capacity (VC: 11–12%) and forced expiratory volume in 1 s (FEV1: about 23%)^29^. Breathing exercises also reduced cravings when incorporated into a 6-month smoking cessation program^30^, while deep breathing significantly improved lung functions^31,32^. Despite the benefits described in these studies, there are numerous gaps in the literature regarding the benefits of a targeted RMT program among persons who smoke, such as the longitudinal effects of RMT on respiratory symptoms, exercise performance, and cessation outcomes.

Even among respondents who reported LB, there was strong interest in a home-based RMT program, which may be interpreted as a heightened awareness regarding lung damage from smoking or a ‘preventive’ approach to minimize known or unknown changes to respiratory health. More recent work also highlights that smokers may be more likely to quit when made aware of their lung health via regular spirometry, even when asymptomatic^33,34^. Recognition of this phenomenon lends support to an intervention program like RMT in conjunction with cessation assistance.

The next steps in substantiating the feasibility and efficacy of home-based RMT among smokers interested in quitting needs to focus on establishing feasibility. While this initial investigation revealed the high level of interest, this was based on responses to a single question. It is also crucial to evaluate individuals’ receptiveness to, and engagement in, the program once implemented. This includes their willingness to adopt the training regimen, perceived barriers to participation (such as physical limitations and/or lack of resources), and potential motivators. During a feasibility study, researchers should monitor engagement by tracking the extent to which participants actively adhere to the prescribed activities. High engagement levels suggest a well-received program, whereas low engagement might indicate areas that need refinement. Researchers should also address implementation feasibility, assessing the practicality of delivering the program on a wider scale, considering factors such as the cost of resources, the training required, and the technological infrastructure needed for home-based delivery. A feasibility study of RMT training among people who smoke cigarettes and are interested in quitting can also provide preliminary estimates of participant satisfaction, changes in symptom severity, quality of life, exercise tolerance, and physical activity.

### Strengths and limitations

Strengths of the study include the diversity of the sample, the low percentage of callers who opted out, and the ability to remotely engage people at high-risk for dyspnea related to current smoking status. Limitations of this study include reliance on a convenience sample and a single-item, Likert-type question assessing level of interest. Respondents were not provided with any further context regarding the frequency/length of training sessions, duration of use, or program costs. Additionally, the study’s limitations include the absence of confounding statistical analysis and limited generalizability to populations across different countries or racial groups. Future studies should provide more information with respect to the details of the RMT program, enroll participants, and determine the impact on dyspnea using a longitudinal design.

## CONCLUSIONS

Our preliminary study revealed that 20% of quitline callers experience clinically significant breathlessness that limits daily activities. Notably, a substantial majority of respondents expressed strong interest in a home-based RMT program, regardless of their breathlessness level. This highlights a promising opportunity to incorporate RMT into smoking cessation support. The next steps will involve rigorously testing the feasibility of implementing this home-based program within our diverse quitline caller population to evaluate engagement, acceptability, and practicality.

## Data Availability

The data supporting this research are available from the authors on reasonable request.

## References

[1] Rosi E, Scano G. Cigarette smoking and dyspnea perception. Tob Induc Dis. 2004;2(March):35-42. doi:10.1186/1617-9625-2-1-35 19570269 PMC2671519

[2] Dyspnea. Mechanisms, assessment, and management: a consensus statement. American Thoracic Society. Am J Respir Crit Care Med. 1999;159(1):321-340. doi:10.1164/ajrccm.159.1.ats898 9872857

[3] Pesola GR, Ahsan H. Dyspnea as an independent predictor of mortality. Clin Respir J. 2016;10(2):142-152. doi:10.1111/crj.12191 25070878 PMC4309743

[4] Woodruff PG, Barr RG, Bleecker E, et al. Clinical significance of symptoms in smokers with preserved pulmonary function. N Engl J Med. 2016;374(19):1811-1821. doi:10.1056/NEJMoa1505971 27168432 PMC4968204

[5] Regan EA, Lynch DA, Curran-Everett D, et al. Clinical and radiologic disease in smokers with normal spirometry. JAMA Intern Med. 2015;175(9):1539-1549. doi:10.1001/jamainternmed.2015.2735 26098755 PMC4564354

[6] Elbehairy AF, Guenette JA, Faisal A, et al. Mechanisms of exertional dyspnoea in symptomatic smokers without COPD. Eur Respir J. 2016;48(3):694-705. doi:10.1183/13993003.00077-2016 27492828

[7] Sheng H, Zhang Y, Shi X, et al. Functional, ultrastructural, and transcriptomic changes in rat diaphragms with different durations of cigarette smoke exposure. Int J Chron Obstruct Pulmon Dis. 2020;15:3135-3145. doi:10.2147/COPD.S278327 33299306 PMC7721115

[8] Nucci RAB, de Souza RR, Suemoto CK, et al. Cigarette smoking impairs the diaphragm muscle structure of patients without respiratory pathologies: an autopsy study. Cell Physiol Biochem. 2019;53:648-655. doi:10.33594/000000163 31556254

[9] Ramon MA, Ter Riet G, Carsin AE, et al. The dyspnea-inactivity vicious circle in COPD: development and external validation of a conceptual model. Eur Respir J. 2018;52(3):1800079. doi:10.1183/13993003.00079-2018 30072504

[10] Ammous O, Feki W, Lotfi T, et al. Inspiratory muscle training, with or without concomitant pulmonary rehabilitation, for chronic obstructive pulmonary disease (COPD). Cochrane Database Syst Rev. 2023;1(1):CD013778. doi:10.1002/14651858.CD013778.pub2 36606682 PMC9817429

[11] Azambuja ACM, de Oliveira LZ, Sbruzzi G. Inspiratory muscle training in patients with heart failure: What is new? Systematic review and meta-analysis. Phys Ther. 2020;100(12):2099-2109. doi:10.1093/ptj/pzaa171 32936904

[12] Illi SK, Held U, Frank I, Spengler CM. Effect of respiratory muscle training on exercise performance in healthy individuals: a systematic review and meta-analysis. Sports Med. 2012;42(8):707-724. doi:10.1007/BF03262290 22765281

[13] Langer D, Ciavaglia C, Faisal A, et al. Inspiratory muscle training reduces diaphragm activation and dyspnea during exercise in COPD. J Appl Physiol (1985). 2018;125(2):381-392. doi:10.1152/japplphysiol.01078.2017 29543134

[14] Bostanci Ö, Mayda H, Yılmaz C, Kabadayı M, Yılmaz AK, Özdal M. Inspiratory muscle training improves pulmonary functions and respiratory muscle strength in healthy male smokers. Respir Physiol Neurobiol. 2019;264:28-32. doi:10.1016/j.resp.2019.04.001 30953791

[15] Cummins SE, Bailey L, Campbell S, Koon-Kirby C, Zhu SH. Tobacco cessation quitlines in North America: a descriptive study. Tob Control. 2007;16(suppl 1):i9-i15. doi:10.1136/tc.2007.020370 18048639 PMC2598516

[16] Fiore MC, Baker TB. Ten million calls and counting: progress and promise of tobacco quitlines in the U.S. Am J Prev Med. 2021;60(3)(suppl 2):S103-S106. doi:10.1016/j.amepre.2020.06.021 33663696 PMC8189745

[17] Hacker KA, Kang JY. Tobacco cessation quitlines: an evolving mainstay for an enduring cessation support infrastructure. Am J Prev Med. 2021;60(3)(suppl 2):S185-S187. doi:10.1016/j.amepre.2020.11.001 33663706

[18] Kessler RC, Andrews G, Colpe LJ, et al. Short screening scales to monitor population prevalences and trends in non-specific psychological distress. Psychol Med. 2002;32(6):959-976. doi:10.1017/s0033291702006074 12214795

[19] Prochaska JJ, Sung HY, Max W, Shi Y, Ong M. Validity study of the K6 scale as a measure of moderate mental distress based on mental health treatment need and utilization. Int J Methods Psychiatr Res. 2012;21(2):88-97. doi:10.1002/mpr.1349 22351472 PMC3370145

[20] Streck JM, Weinberger AH, Pacek LR, Gbedemah M, Goodwin RD. Cigarette smoking quit rates among persons with serious psychological distress in the United States from 2008 to 2016: are mental health disparities in cigarette use increasing? Nicotine Tob Res. 2020;22(1):130-134. doi:10.1093/ntr/nty227 30351429 PMC8360615

[21] Cook BL, Wayne GF, Kafali EN, Liu Z, Shu C, Flores M. Trends in smoking among adults with mental illness and association between mental health treatment and smoking cessation. JAMA. 2014;311(2):172-182. doi:10.1001/jama.2013.284985 24399556 PMC5555156

[22] Fletcher CM, Elmes PC, Fairbairn AS, Wood CH. The significance of respiratory symptoms and the diagnosis of chronic bronchitis in a working population. Br Med J. 1959;2(5147):257-266. doi:10.1136/bmj.2.5147.257 13823475 PMC1990153

[23] Bestall JC, Paul EA, Garrod R, Garnham R, Jones PW, Wedzicha JA. Usefulness of the Medical Research Council (MRC) dyspnoea scale as a measure of disability in patients with chronic obstructive pulmonary disease. Thorax. 1999;54(7):581-586. doi:10.1136/thx.54.7.581 10377201 PMC1745516

[24] Mahler DA, Wells CK. Evaluation of clinical methods for rating dyspnea. Chest. 1988;93(3):580-586. doi:10.1378/chest.93.3.580 3342669

[25] Munari AB, Gulart AA, Dos Santos K, Venâncio RS, Karloh M, Mayer AF. Modified medical research council dyspnea scale in GOLD classification better reflects physical activities of daily living. Respir Care. 2018;63(1):77-85. doi:10.4187/respcare.05636 28874609

[26] Mahler DA, Ward J, Waterman LA, McCusker C, ZuWallack R, Baird JC. Patient-reported dyspnea in COPD reliability and association with stage of disease. Chest. 2009;136(6):1473-1479. doi:10.1378/chest.09-0934 19696126 PMC3026583

[27] Agustí A, Celli BR, Criner GJ, et al. Global initiative for chronic obstructive lung disease 2023 report: GOLD executive summary. Eur Respir J. 2023;61(4):2300239. doi:10.1183/13993003.00239-2023 36858443 PMC10066569

[28] Jones PW, Adamek L, Nadeau G, Banik N. Comparisons of health status scores with MRC grades in COPD: implications for the GOLD 2011 classification. Eur Respir J. 2013;42(3):647-654. doi:10.1183/09031936.00125612 23258783

[29] Jun HJ, Kim KJ, Nam KW, Kim CH. Effects of breathing exercises on lung capacity and muscle activities of elderly smokers. J Phys Ther Sci. 2016;28(6):1681-1685. doi:10.1589/jpts.28.1681 27390394 PMC4932035

[30] Klinsophon T, Thaveeratitham P, Janwantanakul P. The effect of three-part breathing exercise on smoking cessation: a 6-month cluster-randomized clinical trial. J Bodyw Mov Ther. 2022;32:156-162. doi:10.1016/j.jbmt.2022.04.015 36180143

[31] Abid N, Rao AR, Babar MN, Ansari M, Awan WA. Effect of deep breathing exercises in healthy smokers: a pilot study. J Pak Med Assoc. 2020;70(7):1209-1213. doi:10.5455/JPMA.16551 32799275

[32] Seo K, Park SH, Park K. Effects of diaphragm respiration exercise on pulmonary function of male smokers in their twenties. J Phys Ther Sci. 2015;27(7):2313-2315. doi:10.1589/jpts.27.2313 26311972 PMC4540870

[33] Martín-Luján F, Santigosa-Ayala A, Pallejà-Millán M, et al. Effectiveness of the spirometry-based motivational intervention to quit smoking: RESET randomised trial. Eur J Gen Pract. 2023;29(1):2276764. doi:10.1080/13814788.2023.2276764 37933978 PMC10631381

[34] Rodriguez-Alvarez MDM, Roca-Antonio J, Martínez-González S, et al. Spirometry and smoking cessation in primary care: the ESPIROTAB STUDY, a randomized clinical trial. Int J Environ Res Public Health. 2022;19(21):14557. doi:10.3390/ijerph192114557 36361437 PMC9658367

